# Non-optimal effectiveness of convalescent plasma transfusion and hydroxychloroquine in treating COVID-19: a case report

**DOI:** 10.1186/s12985-020-01354-6

**Published:** 2020-06-19

**Authors:** Tian-min Xu, Bin Lin, Cong Chen, Long-gen Liu, Yuan Xue

**Affiliations:** 1 Department of Infectious Diseases, the Third People’s Hospital of Changzhou, Changzhou, Jiangsu China; 2 Department of Infectious Diseases, the First People’s Hospital of Jintan, Changzhou, Jiangsu China; 3 Changzhou Center for Disease Control and Prevention, Changzhou, Jiangsu China; 4 Institute of Hepatology, the Third People’s Hospital of Changzhou, No. 300 Lanling North Road, Changzhou, 213000 Jiangsu China

**Keywords:** COVID-19, SARS-CoV-2, Convalescent plasma, Hydroxychloroquine, Cycle threshold

## Abstract

**Background:**

Convalescent plasma (CP) transfusion was reported to be effective in treating critically ill patients with COVID-19, and hydroxychloroquine could potently inhibit SARS-CoV-2 in vitro. Herein, we reported a case receiving combination therapy with CP transfusion and hydroxychloroquine for the first time.

**Case presentation:**

Laboratory findings showed high lactic acid level (2.1 mmol/L) and C-reactive protein (CRP, 48.8 mg/L), and low white blood cell count (1.96 × 10^9^/L) in a 65-year-old Chinese man, who was diagnosed with severe COVID-19. CP was intravenously given twice, and hydroxychloroquine was orally administrated for a week (0.2 g, three times a day). The lactic acid and C-reactive protein levels remained high (2.1 mmol/L and 73.23 mg/L, respectively), while the arterial oxyhemoglobin saturation decreased to 86% with a low oxygenation index (OI, 76 mmHg) on day 4 after CP transfusion. His temperature returned to normal and the OI ascended above 300 on day 11. Moreover, the RNA test remained positive in throat swab, and computed tomography revealed severe pulmonary lesions on day 11 after admission.

**Conclusion:**

These findings suggested that the effectiveness of combination therapy with CP and hydroxychloroquine may be non-optimal, and specific therapy needs to be explored.

## Background

Coronavirus disease 2019 (COVID-19), caused by severe acute respiratory syndrome coronavirus 2 (SARS-CoV-2), is a pandemic that is rapidly spreading worldwide. It has been reported that 10–20% of severe patients with mild atypical symptoms initially, can rapidly progress to acute respiratory distress syndrome, the main cause of respiratory failure [1].

Few studies have reported the effectiveness of convalescent plasma (CP) transfusion in treating critically ill patients with COVID-19 [2–4]. The clinical symptoms were significantly improved within 3 days, and the viral load was undetectable within 7 days after CP transfusion [3]. These results are encouraging and worthy of further investigation. Nevertheless, Zeng et al. [5] reported that SARS-CoV-2 became undetectable after CP transfusion, but it could not reduce mortality in critically ill COVID-19 patients.

Moreover, hydroxychloroquine was found to be active against SARS-CoV-2 in vitro, and is a treatment option in clinical practice. Yao et al. [6] reported that hydroxychloroquine was more potent than chloroquine to inhibit SARS-CoV-2 in vitro.

Herein, we administered CP transfusion and hydroxychloroquine to a patient with severe COVID-19, and analyzed their clinical symptoms, oxygenation index (OI), and dynamics of viral load.

## Case presentation

A 65-year-old Chinese man, who was diagnosed with laboratory-confirmed COVID-19, was admitted to our isolation ward on 25 March 2020. A week before his admission, he flew in from Brazil airport, and was subsequently under home isolation. On 23 March 2020, he presented with fever (38.8 °C), chills and myalgia. Viral RNA test from throat swab was positive. The cycle threshold (Ct) values of open reading frame 1ab (*Orf1ab*) and nucleocapsid (*N*) genes by RT-PCR assay were 20 and 21, indicating a high viral load. Computed tomography (CT) scan showed bilateral pneumonia. Laboratory findings showed high lactic acid level (2.1 mmol/L) and C-reactive protein (CRP, 48.8 mg/L), and low white blood cell count (1.96 × 10^9^/L), indicative of severe COVID-19. Oxygen and atomized inhalation of recombinant human interferon-ɑ2b was given at admission. CP from two convalescent patients was intravenously given, and hydroxychloroquine (Shanghai Zhongxi Pharmaceuticals, Shanghai, China) was orally administrated for a week (0.2 g, three times a day).

**Fig. 1:**
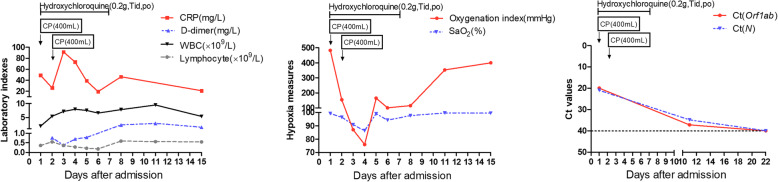
Dynamics of laboratory indexes in the patient with severe COVID-19 receiving convalescent plasma and hydroxychloroquine treatment. CP, convalescent plasma; CRP, C-reactive protein; WBC, white blood cells; SaO2, oxyhemoglobin saturation; Ct, cycle threshold

**Fig. 2:**
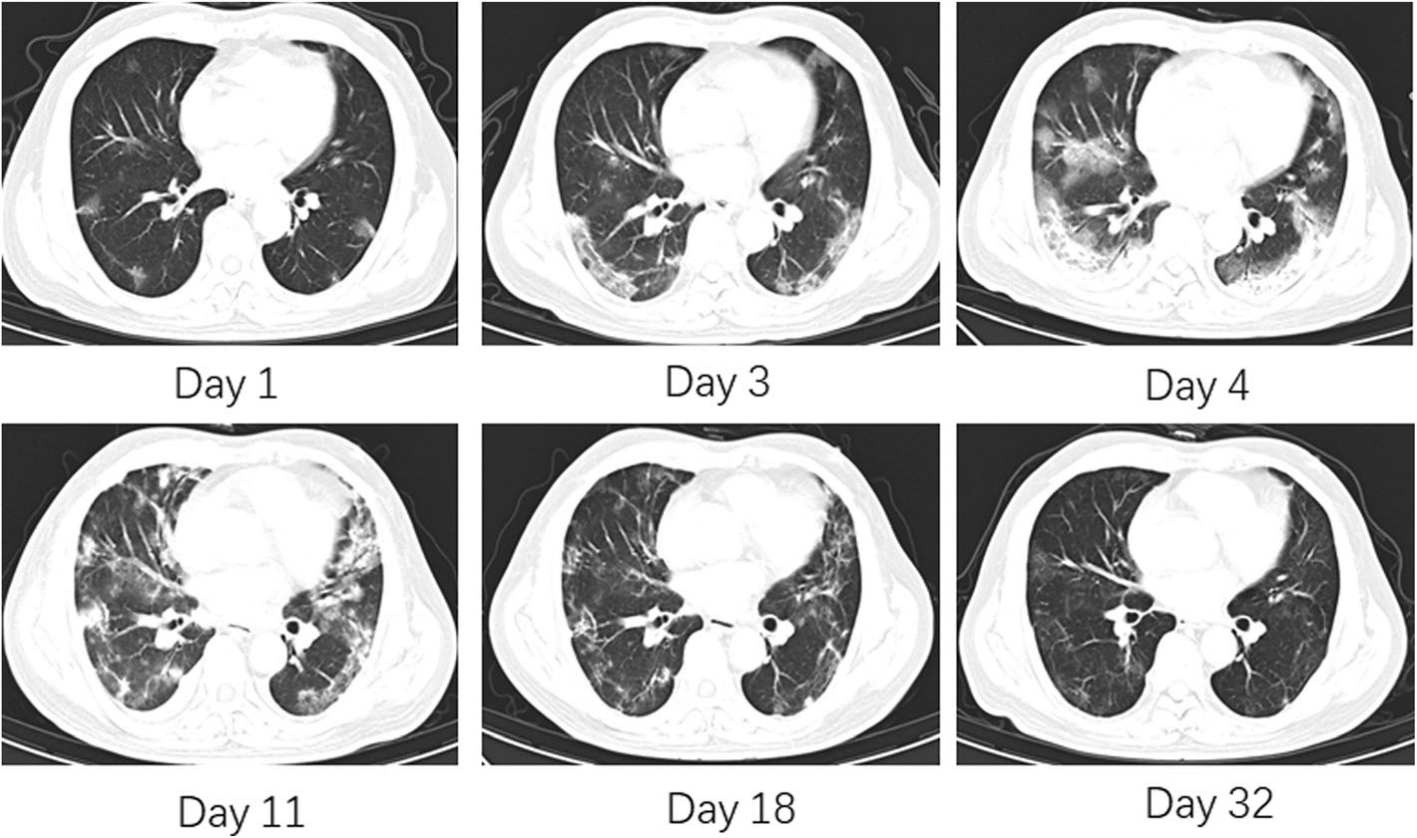
Dynamics of computed tomography scans after admission

As shown in Fig. 1, on day 4 after CP transfusion, the lactic acid and CRP levels remained high (2.1 mmol/L and 73.23 mg/L, respectively). The arterial oxyhemoglobin saturation (SaO_2_) decreased to 86%, while the OI decreased to 76 mmHg, and mechanical ventilation was administered. His temperature returned to normal and the OI ascended above 300 on day 11, after which the ventilator was withdrawn. On day 11 after CP transfusion, the RNA test remained positive in throat swab, and CT revealed severe pulmonary lesions (Fig. 2).

In addition, no apparent side-effects were found during CP transfusion and hydroxychloroquine treatments.

## Discussion and conclusion

Currently, the pathogenesis of COVID-19 remains unclear, and no specific treatment is available. Cytokine storm and excessive inflammation coexist and may lead to multiple organ failure. Both antiviral therapy and treatment of the systematic response are important for patients with rapid deterioration of COVID-19.

In a study with small sample size, the median time from onset of illness to CP transfusion was 16.5 days, and the disappearance of viremia was observed in 7 days after CP transfusion [3]. The results suggested that CP therapy could potentially improve the clinical outcomes by neutralizing viremia in severe COVID-19 [3]. In another study, CP transfusion was given at a median of 21.5 days after first detection of SARS-CoV-2, the viral load became negative 3 days after CP transfusion, though 5 of 6 patients died eventually [5]. Unexpectedly, the viral load remained detectable on day 11 after combination therapy with CP and hydroxychloroquine. Considering the time from onset of illness to CP transfusion in previous studied [3, 5], it is difficult to prove whether the viremia is cured by the CP and antiviral agent, or is a natural course of COVID-19. Indeed, there is a limitation that antibodies against SARS-CoV-2 in CP from the two convalescent patients and the present patient are not detected. Based on our clinical experience, sufficient evidence supporting the use of CP and hydroxychloroquine in treating COVID-19 is lacking.

Our findings suggested that the effectiveness of combination therapy with CP and hydroxychloroquine may be non-optimal, and specific therapy needs to be explored.

## Data Availability

Derived data are available on reasonable request.

## Abbreviations

CP: Convalescent plasma
COVID-19: Coronavirus disease 2019
SARS-CoV-2: Severe acute respiratory syndrome coronavirus 2
OI: Oxygenation index
Ct: Cycle threshold
*Orf1ab*: Open reading frame 1ab
CT: Computed tomography
CRP: C-reactive protein
